# Quantification of Bacteria in Mouth-Rinsing Solution for the Diagnosis of Periodontal Disease

**DOI:** 10.3390/jcm10040891

**Published:** 2021-02-22

**Authors:** Jeong-Hwa Kim, Jae-Woon Oh, Young Lee, Jeong-Ho Yun, Seong-Ho Choi, Dong-Woon Lee

**Affiliations:** 1 Department of Periodontology, Dental Hospital, Veterans Health Service Medical Center, Seoul 05368, Korea; choco1206@naver.com (J.-H.K.); ohsoon1052@naver.com (J.-W.O.); 2 Veterans Medical Research Institute, Veterans Health Service Medical Center, Seoul 05368, Korea; lyou7688@gmail.com; 3 Department of Periodontology, College of Dentistry and Institute of Oral Bioscience, Jeonbuk National University, Jeonju 54896, Korea; grayheron@hanmail.net; 4 Department of Periodontology, College of Dentistry and Research Institute for Periodontal Regeneration, Yonsei University, Seoul 03722, Korea; SHCHOI726@yuhs.ac

**Keywords:** polymerase chain reaction, periodontitis, bacteria

## Abstract

This study aimed to evaluate the feasibility of diagnosing periodontitis via the identification of 18 bacterial species in mouth-rinse samples. Patients (n = 110) who underwent dental examinations in the Department of Periodontology at the Veterans Health Service Medical Center between 2018 and 2019 were included. They were divided into healthy and periodontitis groups. The overall number of bacteria, and those of 18 specific bacteria, were determined via real-time polymerase chain reaction in 92 mouth-rinse samples. Differences between groups were evaluated through logistic regression after adjusting for sex, age, and smoking history. There was a significant difference in the prevalence (healthy vs. periodontitis group) of *Aggregatibacter actinomycetemcomitans* (2.9% vs. 13.5%), *Treponema denticola* (42.9% vs. 69.2%), and *Prevotella nigrescens* (80% vs. 2.7%). Levels of *Treponema denticola*, *Prevotella nigrescens*, and *Streptococcus mitis* were significantly associated with severe periodontitis. We demonstrated the feasibility of detecting periopathogenic bacteria in mouth-rinse samples obtained from patients with periodontitis. As we did not comprehensively assess all periopathogenic bacteria, further studies are required to assess the potential of oral-rinsing solutions to indicate oral infection risk and the need to improve oral hygiene, and to serve as a complementary method for periodontal disease diagnosis.

## 1. Introduction

Subgingival plaque bacteria are the main etiology of periodontitis. Complex interactions between certain pathogens are key in the development of periodontal disease [1]. Microbial complexes in the subgingival biofilm are classified into five groups: red, green, orange, yellow, and purple. In particular, the red group, which is composed of *Tannerella forsythia, Treponema denticola,* and *Porphyromonas gingivalis,* has been determined as one of the main causes of periodontal disease [1].

Several studies have shown that the presence and number of these bacteria are related to disease prediction criteria such as probing depth, bone loss, attachment loss, and bleeding on probing [2,3]. Various bacterial species besides those of the red complex have been found to be key in the development and progression of periodontitis; among these species, *P. gingivalis, Prevotella intermedia*, and *Aggregatibacter actinomycetemcomitans* have been shown to have the strongest association with periodontal disease [4]. A previous study showed that *P. gingivalis, T. denticola,* and *A. actinomycetemcomitans*, when present in saliva, contributed to pocket deepening [5]. In order to detect the bacteria associated with periodontal disease, plaque is usually collected from a specific tooth and analyzed [4,6]. Most studies have analyzed bacterial groups using plaque samples [7].

Multiplex real time-polymerase chain reaction (RT-PCR) allows RT measurement of amplified deoxyribonucleic acid (DNA) using a fluorescent substance. In general PCR, the final product is observed via agarose gel electrophoresis; therefore, accurate bacterial quantification is impossible. However, multiplex RT-PCR can be used to quantitatively analyze the product amplified per PCR cycle.

Mouth-rinsing solutions have been used in various sialochemistry studies [8,9]. Recently, some studies assessing the prevalence and levels of specific bacterial species have been conducted using PCR analysis of mouth-rinsing solutions [10]. However, very few studies have investigated the link between the diagnosis of periodontitis and the oral bacteria present in a mouth-rinsing solution. Additionally, the phosphate-buffered saline solution used in previous studies has been reported to cause discomfort.

The purpose of this study was to examine the correlation between periodontal disease and 18 different bacteria by conducting a RT-PCR analysis of mouth-rinsing solutions and to evaluate the usefulness of this diagnostic method.

## 2. Materials and Methods

### 2.1. Patient Selection

Patients who visited the Department of Periodontology at the Veterans Health Service Medical Center between 2018 and 2019 for various reasons underwent routine examination. Due to the lack of prior studies conducted with rinsing solutions, we decided to use this method to compare bacterial species prevalence and levels in healthy patients and patients with severe periodontal disease. After examination, 110 patients were selected to participate in the study.

**Figure 1:**
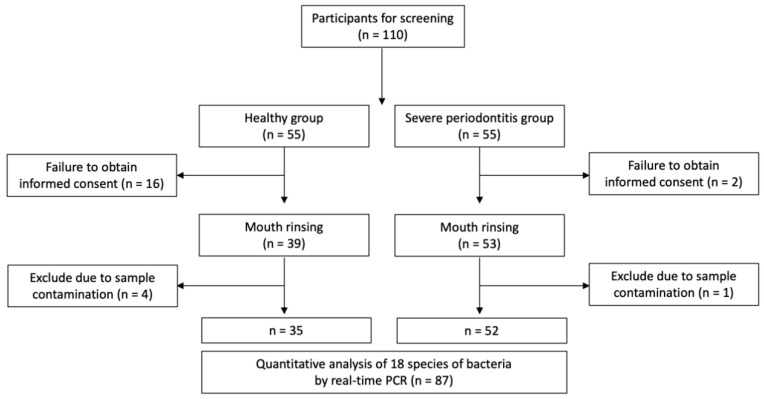
Study flow chart.

However, 18 patients refused to participate in the mouth-rinsing test. Hence, 92 patients were finally included in this study. Additionally, five subjects were excluded from the study because their mouth-rinsing solutions were contaminated in the process of transferring the collected samples (Figure 1). The study protocol was approved by the institutional review board of Veterans Health Service Medical Center (BOHUN No. 2018-03-002). All participants provided written informed consent. This study was conducted according to the Helsinki Declaration of 1975 and its later revisions.

### 2.2. Sample Size Determination

The sample size was calculated using G*Power 3.1 software [11]. Comparisons between the two groups were conducted at a two-sided alpha level of 5% and a power of 90%. It was determined that a sample size of 42 participants per group would provide a power of 90% for the detection of between-group differences. However, considering a drop-out rate of 25%, a sample size of 55 patients per group was finalized.

### 2.3. Periodontal Examination

Each patient underwent an assessment of the probing depth and gingival recession at six sites per tooth using a periodontal probe (PCP-12, Hu-Friedy, Rotterdam, The Netherlands) by one examiner. Attachment loss was also measured.

After the dental examination, the presence and severity of periodontal disease were determined according to the Centers for Disease Control and Prevention/American Academy of Periodontology definitions [12]. We performed an additional examination using a mouth-rinse solution in both healthy patients and those with severe periodontal disease. Severe periodontitis was defined as two or more interproximal sites with a clinical attachment loss ≥ 6 mm, which are not the same area, and one or more interproximal sites with a probing depth ≥ 5 mm.

### 2.4. Sample Collection and DNA Extraction

Mouth-rinse samples were collected in the morning after regular brushing. Each subject rinsed their mouth with 10 mL of Easygen gargle (YD Global Life Science, Seongnam, Korea) for 60 s, after which the gargling liquid containing the patient’s saliva was collected as previously described [13]. DNA was extracted from the gargle sample using a Qiagen column (DNA Mini Kit, Qiagen, Hilden, Germany), according to the manufacturer’s instructions.

### 2.5. Multiplex Quantitative RT-PCR (qPCR)

The qPCR was performed with the EasyPerio molecular kit (YD Global Lifescience, Seongnam, Korea), according to the manufacturer’s instructions. The kit consisted of 8 different oligo mixes and 2 × master mixes. This was designed according to the typical multiplex qPCR method [14]. The CFX96 Touch™ RT-PCR Detection System (Bio-Rad, Hercules, CA, USA) was used for qPCR. The sequential steps in the PCR procedure were as follows: pre-denaturation for 30 s at 95 °C; 40 cycles of 5 s denaturation at 95 °C; and 30 s extension and annealing at 62 °C. Fluorescence scanning was performed after the extension and annealing step. Information on the primers and probes is displayed in Table 1. In this way, DNA of 18 species of bacteria was extracted and analyzed by RT-qPCR. The 18 species of bacteria were the following: *A. actinomycetemcomitans*, *P. gingivalis*, *T. forsythia*, *T. denticola*, *Fusobacterium nucleatum*, *P. intermedia*, *Parvimonas micra*, *Campylobacter rectus*, *Eubacterium nodatum*, *Eikenella corrodens*, *Streptococcus mitis*, *Streptococcus mutans*, *Lactobacillus casei*, *Staphylococcus aureus*, *Enterococcus faecalis*, *Actinomyces viscosus*, *Prevotella nigrescens*, and *Streptococcus sobrinus*.

### 2.6. Bacterial Quantification

Standard curves were generated using the 18 plasmids at five different concentrations. The plasmids’ DNA contained specific sequences of each microorganism. Each bacterial gene used for plasmid construction is listed in Table 1. The copy numbers of each oral-bacterial DNA were calculated by substituting the cycle threshold values obtained from the qPCR into the quantitative formula obtained through the standard curve.

### 2.7. Statistical Analysis

This study evaluated whether there was a significant difference in the prevalence and levels of bacterial species between healthy individuals and those with periodontitis. Sex and smoking history were expressed as frequencies and percentages, and age, as means and standard deviations. The total number of bacteria was reported as median and interquartile range, and the number of each bacterial species was reported after normalization (dividing by the total number of bacteria in each sample). Differences in prevalence between groups were evaluated through logistic regression. Spearman’s rank correlation was used to examine the association between the levels of the different target species. Only two species that had at least five complete observations were estimated with the correlation coefficient. Logistic regression models were applied with disease status (healthy or with periodontal disease) as the dependent variable and the bacterial category as the independent variable. The bacterial category comprised three levels. Level 0 represented PCR-negative subjects, while levels 1 and 2 were categorized according to the median of the number of bacterial cells in PCR-positive subjects; levels 1 and 2 were assigned to values less than or greater than the median, respectively.

The Firth’s penalized maximum-likelihood bias-reduction method was used to estimate the odds ratio when there was a complete separation [27,28]. All regression analyses were adjusted for known confounders of periodontitis, including age, sex, and smoking history.

Statistical analyses were performed using R 3.5.1 (R Development Core Team; R Foundation for Statistical Computing, Vienna, Austria). *p* values < 0.05 were considered statistically significant.

## 3. Results

**Figure 2:**
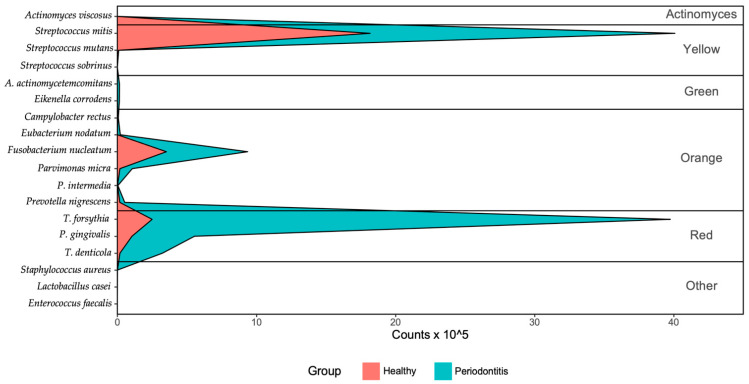
Mean bacterial cells in the healthy group and periodontal disease group.

There were 35 individuals in the healthy group and 52 in the severe periodontitis group (Table 2). Figure 2 shows the mean counts of bacteria in the two groups. The number of bacteria of the red, yellow, and orange groups was higher in patients with periodontal disease than in the healthy group. The results of the quantitative analysis of the 18 species of bacteria are shown in Table 3. *S. mitis*, *P. micra*, and *F. nucleatum* were found in all subjects in the healthy group. *P. nigrescens* and *C. rectus* were found in 80% of the subjects in the healthy group. Among bacteria in the red complex group, *P. gingivalis* was found in 45.7%, *T. forsythia* in 74.3%, and *T. denticola* in 42.9% of the subjects in the healthy group. *E. faecalis* and *A. viscosus* were not detected in any of the healthy subjects. Similar to the healthy group, *S. mitis*, *P. micra*, and *F. nucleatum* were found in all subjects in the severe periodontitis group. *P. gingivalis*, *P. nigrescens*, *T. forsythia*, and *T. denticola* were detected in 90.4%, 82.7%, 73.1%, and 69.2% of individuals with severe periodontitis, respectively. After adjusting for age, sex, and smoking history, there were differences in the prevalence of *A. actinomycetemcomitans*, *T. denticola*, and *P. nigrescens* between the healthy group and severe periodontitis group. Among the red complex group bacteria, only *T. denticola* prevalence was significantly different between groups (Table 3).

Table 4 shows the correlations between the different bacterial species in all participants. Correlation coefficients ranged from −1 to 1, with numbers greater than 0 indicating positive correlations and numbers lower than 0 indicating negative correlations. *A. actinomycetemcomitans* and *P. gingivalis* showed a correlation of 0.96 and a *p* value lower than 0.05, indicating a significant positive correlation. *P. gingivalis* had a positive correlation with *E. nodatum* and a negative correlation with *S. mitis. T. forsythia* was negatively correlated with *F. nucleatum*, *P. nigrescens*, and *S. mitis*. *F. nucleatum* was positively correlated with *P. nigrescens*, *S. mitis*, and *L. casei*. *P. intermedia* was positively correlated with *P. nigrescens* and *C. rectus*.

Table 5 shows the correlations between bacterial species in the healthy group. *T. forsythia* was positively correlated with *C. rectus* and negatively correlated with *S. mitis. T. denticola* was negatively correlated with *S. mitis.*

Table 6 shows the correlations between bacterial species in the periodontal disease group. *P. gingivalis* had a significant positive correlation with *A. actinomycetemcomitans*. *F. nucleatum* was negatively correlated with *T. forsythia.*

Table 7 shows the categorization of the number of bacteria into three levels. *P. gingivalis*, *T. denticola*, *P. micra*, *S. mitis*, *L. casei*, *S. aureus*, *E. nodatum*, and total bacteria were significantly associated with severe periodontitis at certain levels. However, after adjusting for factors such as sex, age, and smoking, only *T. denticola*, *P. nigrescens*, and *S. mitis* were significant. *T. denticola* significance was only noted at level 2, in which the risk of periodontal disease was 7.3 times higher compared to level 0. *P. nigrescens* was significantly associated with severe periodontitis at levels 1 and 2; the risk of periodontal disease at level 2 was 22.5 times higher than that at level 0. *S. mitis* significance was only observed at level 2.

## 4. Discussion

To the best of our knowledge, this preliminary study is the first to quantify bacteria with PCR in a mouth-rinsing solution, as opposed to a subgingival plaque or saliva sample. Newer diagnostic methods have been developed with more detailed stages and grades corresponding to the related treatment protocol [29]. While periodontal probing is the traditional method used for diagnosing periodontal disease, the detection of periopathogenic bacteria with PCR may potentially serve as an adjunct assessment. Nevertheless, to date, no standardized methods have been proposed for the diagnosis of periodontal disease based on gargled solutions [30].

Several studies on periodontal pathogens have been conducted using RT-PCR analysis. *P. gingivalis*, *T. forsythia*, *T. denticola*, and *P. intermedia* have been reported to be mainly prevalent in Asian populations [31,32]. However, *A. actinomycetemcomitans* prevalence varies widely. In this study, a low *A. actinomycetemcomitans* prevalence was observed. Previous studies have reported even lower levels in this and other previous studies compared to other pathogens [2,33]. In line with the results of previous studies, we found significant differences between the groups of bacteria known to be related to periodontal disease. The prevalence of *A. actinomycetemcomitans*, *T. denticola*, *P. nigrescens*, and *S. mitis* were significantly different between the healthy and periodontal disease groups.

*A. actinomycetemcomitans* is a common pathogen in aggressive periodontitis, and it is known to have mutually inhibitory effects on *Streptococcus sanguis*, *Streptococcus uberis*, and *A. viscosus* [34]. *A. actinomycetemcomitans* is involved in the pathogenesis of aggressive periodontitis in younger patients [35]. *T. denticola* and *P. nigrescens* are both known to be related to periodontitis. A previous study showed clear evidence of increased immune responses to *T. denticola*, *P. nigrescens*, and *F. nucleatum* in 89 patients with chronic periodontitis [36]. *F. nucleatum* is frequently detected in the subgingival plaque of patients with chronic periodontitis and is often found associated with periodontal pockets. *A. actinomycetemcomitans*, *T. forsythia*, *T. denticola*, and *P. gingivalis* are strongly associated with periodontal disease, disease progression, and treatment failure. *P. intermedia*, *P. micra, C. rectus*, *E. nodatum*, *P. nigrescens*, and *F. nucleatum* can also act as pathogens if their concentrations exceed certain thresholds [37].

Periodontal disease is a result of complex interactions between the periodontal pathogens and normal flora [38]. This fact rationalizes the use of mouth-rinsing solution for bacterial analysis, as it provides mixed bacterial samples. Nevertheless, the presence of periodontal pathogens in the gingival crevices by itself does not cause or initiate periodontal inflammation. The bacterial load in an area with periodontal disease is higher than that in a healthy area; these bacteria are called periodontopathic [39]. *P. gingivalis* and *T. forsythia* are some of the main pathogens of periodontitis, but no significant difference was found between the healthy and periodontal disease groups in this study. The distribution, as well as the number of bacterial species varies in diseased and healthy periodontal tissues. In this study, *S. mitis*, a Gram-positive strain present in healthy tissues, had a 100% prevalence in both normal and severe periodontitis groups. *F. nucleatum*, which belongs to the red complex group and is strongly associated with periodontal disease, also had a 100% prevalence in both groups. Therefore, although these bacterial species may be proportionally less dominant, they are present in the oral cavity as a constituent of the normal flora [40]. Our findings revealed a significant positive correlation between *A. actinomycetemcomitans* and *P. gingivalis*. This indicates that both bacterial species affect each other’s growth [38]. In addition, *P. gingivalis* and *E. nodatum* also showed a positive correlation, indicating that the higher the number of P. *gingivalis,* the higher the number of *E. nodatum*. Conversely, *T. forsythia* was negatively correlated with *F. nucleatum*, *P. nigrescens*, and *S. mitis*. Hence, these bacteria may inhibit each other’s growth.

After dividing bacterial levels according to whether they were above or below the median, and adjusting for confounding factors (e.g., sex, age, and smoking habit), *T. denticola*, *P. nigrescens*, and *S. mitis* were significantly associated with periodontitis. These results indicate that the risk of periodontal disease is increased if the levels of *T. denticola* and *P. nigrescens* are high. It can also be inferred that the higher the level of *S. mitis*, the lower the risk of developing periodontal disease.

This study has some limitations because we could not verify the reproducibility of our results. Moreover, in order for the mouth-rinsing solution analysis to be of diagnostic value, a certain number of bacteria must be detected to indicate disease. Implementation of the new classification system described above was not possible when recruiting participants in this study. We could only divide participants into two groups: healthy and severe periodontitis. Due to the lack of previous studies on diagnostic methods using mouth-rinsing solutions, we tried to evaluate differences between the two groups using the existing classification method. This should be complemented in the next study. There were limitations in adjusting for age, sex, and smoking history, because of the small sample size. Among the correction variables, age is an important variable related to periodontal disease, but in this study, the sample size was not large enough to consider the correction variable, even though it had already been adjusted.

As mentioned earlier, periodontitis is a disease with various factors caused by subgingival bacterial colonies such as *A. actinomycetemcomitans*, *T. forsythia*, and *P. gingivalis.* [41]. However, *P. gingivalis* was not significantly associated with periodontitis after adjustment for confounding factors in this study. *P. gingivalis* has been shown to have a higher prevalence in deep pockets [1,42,43]. Therefore, the results of our study may reflect the low ability of mouth rinsing to sample *P. gingivalis* in these regions. Because the number of bacteria needed to cause periodontal disease may vary depending on the host’s immune system, additional research methods are needed, such as comparing with crevicular fluid and gingival biopsy to show reliable results [44].

This study suggests that the analysis of mouth-rinsing solution might be a promising diagnostic method, and further studies with greater sensitivity should be conducted with larger samples to determine its perceived usefulness. Diagnosing the severity of periodontitis by analyzing gargled mouth-rinse solutions is less invasive than collecting plaque samples. We hope that the analysis of mouth-rinsing solutions will become an accepted diagnostic method for periodontal disease. A limitation of this study is that only three periopathogenic bacteria, among a total of 18 species, exhibited a significant difference between the healthy and periodontal disease groups; nevertheless, the advantages of the detection method are obvious.

In summary, the findings of this study are as follows: (1) similar to previous studies, bacteria known to cause periodontal disease were detected with mouth-rinsing solutions in patients with severe periodontal disease; (2) significant differences were found in the prevalence (healthy vs. periodontal disease group) of *A. actinomycetemcomitans* (2.9% vs. 13.5%), *T. denticola* (42.9% vs. 69.2%), and *P. nigrescens* (80% vs. 82.7%); and (3) *T. denticola*, *P. nigrescens*, and *S. mitis* levels were significantly different between groups in the quantitative analysis.

We did not comprehensively assess all periopathogenic bacteria in this study; therefore, additional research is required to assess the potential of oral-rinsing solutions to reflect oral-infection risk and the need to improve oral hygiene, as well as to serve as a complementary method for periodontal disease diagnosis. Similar to the results of plaque analysis, which has been conducted in many studies, the results obtained by detecting bacteria in mouth-rinsing solutions show that there is a relationship between specific bacteria and severe periodontal disease. While mouth-rinsing solutions are non-invasive, simple, and capable of detecting a wide range of bacterial species, they are limited by the lack of clear diagnostic criteria. Therefore, in order for this diagnostic method to be effective, research aimed at establishing the criteria for the type and number of bacteria should be conducted. Recently, the concept of the diagnosis of periodontitis has been improved to complement the treatment stage. If this simple diagnostic kit is quantified and developed, it is expected to be helpful in future treatment planning.

## Figures and Tables

**Table 1:** Primers and probes of the 18 species of bacteria analyzed.

Bacteria	Target Gene	Primer/ Probe	Sequence (5′-3′)	Ref.	Bacteria	Target Gene	Primer/ Probe	Sequence (5′-3′)	Ref.
*Aggregatibacter* *actinomycetemcomitans*	leukotoxin	Forward	CG**********GA	[15]	*Eubacterium* *nodatum*	hypothetical protein	Forward	TG**********GA	[16]
		Reverse	AT**********CA				Reverse	AA**********AT	
		Probe	[FAM]GG**********CC[BHQ1]				Probe	[TR]TT**********GG[BHQ2]	
*Porphyromonas* *gingivalis*	hemagglutinin	Forward	AC**********GC	[17]	*Eikenella* *corrodens*	prolineiminopeptidase	Forward	GC**********TG	[16]
		Reverse	GC**********CT				Reverse	GC**********TT	
		Probe	[HEX]CG**********GA[BHQ1]				Probe	[Cy5]AC**********AT[BHQ2]	
*Tannerella* *forsythia*	karilysin protease	Forward	TG**********CC	[18]	*Streptococcus* *mitis*	16S ribosomal RNA	Forward	GT**********CG	[19]
		Reverse	TT**********CA				Reverse	TA**********AT	
		Probe	[TR]CC**********GG[BHQ2]				Probe	[FAM]TA**********CC[BHQ1]	
*Treponema* *denticola*	OpdB	Forward	AG**********AG	[20]	*Streptococcus* *mutans*	PTS EII	Forward	CA**********CA	[21]
		Reverse	GC**********AT				Reverse	TG**********CC	
		Probe	[Cy5]CG**********TC[BHQ2]				Probe	[HEX]TG**********GG[BHQ1]	
*Fusobacterium* *nucleatum*	16S ribosomal RNA	Forward	GG**********TC	[22]	*Streptococcus* *sobrinus*	Ftsk	Forward	GG**********CC	[23]
		Reverse	CT**********GC				Reverse	AC**********GG	
		Probe	[FAM]AA**********CG[BHQ1]				Probe	[TR]AG**********GC[BHQ2]	
*Prevotella* *intermedia*	hemagglutinin	Forward	CA**********AC	[15]	*Lactobacillus* *casei*	att	Forward	CA**********GT	[24]
		Reverse	CA**********TC				Reverse	AC**********CC	
		Probe	[HEX]CC**********AC[BHQ1]				Probe	[Cy5]TG**********GT[BHQ2]	
*Prevotella* *nigrescens*	gyrase subunit B	Forward	AG**********CT	[16]	*Staphylococcus* *aureus*	clumping factor A	Forward	GC**********AA	[25]
		Reverse	GC**********CT				Reverse	GA**********TT	
		Probe	[TR]GC**********AA[BHQ2]				Probe	[FAM]TG**********CA[BHQ1]	
*Parvimonas* *micra*	16S ribosomal RNA	Forward	GA**********AG	[15]	*Enterococcus* *faecalis*	gelE-sprE operon	Forward	GA**********TT	[26]
		Reverse	GG**********CC				Reverse	CG**********AC	
		Probe	[FAM]GG**********CA[BHQ1]				Probe	[HEX]GC**********GA[BHQ1]	
*Campylobacter* *rectus*	GroEL	Forward	AA**********GG	[16]	*Actinomyces* *viscosus*	nanH	Forward	GC**********CG	[21]
		Reverse	TC**********GA				Reverse	GA**********CA	
		Probe	[HEX]GG**********GT[BHQ1]				Probe	[TR]GA**********AA[BHQ2]	

**Table 2:** Participant demographics.

Characteristic		Healthy Group (*n* = 35)	Severe Periodontitis Group (*n* = 52)
Age (Years, mean ± SD)		39.0 ± 17.9	56.2 ± 15.2
Sex	Male	29 (83%)	44 (85%)
	Female	6 (17%)	8 (15%)
Smoking	Non-smokers	31 (89%)	46 (88%)
	Current smokers	4 (11%)	6 (12%)

Abbreviations: SD, Standard deviation.

**Table 3:** Prevalence of target species and their quantities in polymerase chain reaction-positive subjects.

Bacteria	Healthy Group (*n* = 35)	Severe Periodontitis Group (*n* = 52)
*Aggregatibacter actinomycetemcomitans*		
Prevalence, *n* (%) ^a^	1 (2.9)	7 (13.5)
Median bacterial cells proportion (%) (IQR)	0.46 (0.46–0.46)	0.75 (0.46–1.07)
*Porphyromonas gingivalis*		
Prevalence, *n* (%)	16 (45.7)	47 (90.4)
Median bacterial cells proportion (%) (IQR)	3.43 (1.74–5.86)	3.83 (2.27–8.28)
*Tannerella forsythia*		
Prevalence, *n* (%)	26 (74.3)	38 (73.1)
Median bacterial cells proportion (%) (IQR)	3.07 (0.65–7.74)	26.07 (4.49–50.82)
*Treponema denticola*		
Prevalence, *n* (%) ^a^	15 (42.9)	36 (69.2)
Median bacterial cells proportion (%) (IQR)	0.59 (0.18–2.34)	2.91 (1.36–5.39)
*Fusobacterium nucleatum*		
Prevalence, *n* (%)	35 (100.0)	52 (100.0)
Median bacterial cells proportion (%) (IQR)	18.73 (13.31–23.15)	12.89 (7.23–20.02)
*Prevotella intermedia*		
Prevalence, *n* (%)	8 (22.9)	15 (28.8)
Median bacterial cells proportion (%) (IQR)	0.22 (0.05–0.46)	0.11 (0.05–0.19)
*Prevotella nigrescens*		
Prevalence, *n* (%) ^a^	28 (80.0)	43 (82.7)
Median bacterial cells proportion (%) (IQR)	0.73 (0.4–1.94)	0.47 (0.14–1.23)
*Parvimonas micra*		
Prevalence, *n* (%)	35 (100.0)	52 (100.0)
Median bacterial cells proportion (%) (IQR)	0.5 (0.29–0.82)	0.99 (0.45–1.81)
*Campylobacter rectus*		
Prevalence, *n* (%)	28 (80.0)	45 (86.5)
Median bacterial cells proportion (%) (IQR)	0.11 (0.06–0.18)	0.08 (0.04–0.13)
*Eubacterium nodatum*		
Prevalence, *n* (%)	3 (8.6)	14 (26.9)
Median bacterial cells proportion (%) (IQR)	0.21 (0.12–0.27)	0.71 (0.3–1.34)
*Eikenella corrodens*		
Prevalence, *n* (%)	4 (11.4)	15 (28.8)
Median bacterial cells proportion (%) (IQR)	0.07 (0.04–0.45)	0.28 (0.15–0.71)
*Streptococcus mitis*		
Prevalence, *n* (%)	35 (100.0)	52 (100.0)
Median bacterial cells proportion (%) (IQR)	73.72 (63.61–79.49)	59.13 (37.87–70.34)
*Streptococcus mutans*		
Prevalence, *n* (%)	23 (65.7)	35 (67.3)
Median bacterial cells proportion (%) (IQR)	0.03 (0.02–0.1)	0.03 (0.01–0.15)
*Streptococcus sobrinus*		
Prevalence, *n* (%)	1 (2.9)	5 (9.6)
Median bacterial cells proportion (%) (IQR)	0.06 (0.06–0.06)	0 (0–0.01)
*Lactobacillus casei*		
Prevalence, *n* (%)	6 (17.1)	18 (34.6)
Median bacterial cells proportion (%) (IQR)	0.01 (0–0.04)	0 (0–0.01)
*Staphylococcus aureus*		
Prevalence, *n* (%)	15 (42.9)	4 (7.7)
Median bacterial cells proportion (%) (IQR)	0.02 (0.01–0.14)	0.03 (0–0.07)
*Enterococcus faecalis*		
Prevalence, *n* (%)	0 (0.0)	0 (0.0)
Median bacterial cells proportion (%) (IQR)	NA (NA–NA)	NA (NA–NA)
*Actinomyces viscosus*		
Prevalence, *n* (%)	0 (0.0)	0 (0.0)
Median bacterial cells proportion (%) (IQR)	NA (NA–NA)	NA (NA–NA)
Total number of cellsPrevalence, *n* (%)	35 (100.0)	52 (100.0)
Median bacterial cells (IQR)	36,126,518 (16,199,034–92,716,204)	108524910 (69,243,624.5–177,393,988.25)

Abbreviations: IQR, interquartile range; NA, not available. ^a^ Significant difference between groups at *p* < 0.05, analyzed using the logistic regression analysis.

**Table 4:** Interspecies correlations in all subjects.

	Aa	Pg	Tf	Td	Fn	Pi	Pn	Pm	Cr	En	Ec	Sm	Smu	Ss	Lc	Sa	Total
Aa		0.96 *	−0.49	0.3	0.38		0.98 *	0.91 *	0.57			0.07					−0.26
Pg			−0.04	0.25	−0.01	−0.06	0.16	0.23	−0.02	0.57 *	−0.3	−0.34 *	0.16	0.5	−0.05	−0.27	−0.06
Tf				0.19	−0.56 *	−0.21	−0.28 *	0.03	−0.02	0.1	−0.37	−0.87 *	−0.22	−0.54	−0.2	−0.21	0.14
Td					−0.17	0.53 *	0.05	0.61 *	−0.08	0.8 *	−0.24	−0.42 *	−0.02	−0.17	0.34	−0.4	−0.07
Fn						0.21	0.25 *	−0.15	0.18	−0.17	0.07	0.25 *	0.02	0.9 *	0.52 *	0.02	−0.13
Pi							0.53 *	0.14	0.51 *	0.51		0.09	−0.09		0.43		0.32
Pn								0.15	0.23	−0.16	−0.13	0.07	−0.18	0.75	−0.03	−0.32	−0.21
Pm									0.18	0.13	−0.27	−0.23 *	0	0.17	−0.02	−0.23	−0.02
Cr										−0.31	−0.24	0.03	−0.15	0.88 *	0.06	−0.24	−0.16
En												−0.13	0.35		0.3		0.42
Ec												0.24	0.19		−0.17		−0.16
Sm													0.12	0.33	0.04	0.23	−0.03
Smu														−0.12	−0.13	−0.15	−0.09
Ss																	−0.62
Lc																	−0.16
Sa																	−0.21
Total																	

Abbreviations: Aa, *Aggregatibacter actinomycetemcomitans*; Pg, *Porphyromonas gingivalis*; Tf, *Tannerella forsythia*; Td, *Treponema denticola*; Fn, *Fusobacterium nucleatum*; Pi, *Prevotella intermedia*; Pm, *Parvimonas micra*; Cr, *Campylobacter rectus*; En, *Eubacterium nodatum*; Ec, *Eikenella corrodens*; Sm, *Streptococcus mitis*; Smu, *Streptococcus mutans*; Lc, *Lactobacillus casei*; Sa, *Staphylococcus aureus*; Pn, *Prevotella nigrescens*; Ss, *Streptococcus sobrinus*; and * *p* < 0.05.

**Table 5:** Interspecies correlations in healthy subjects.

	Aa	Pg	Tf	Td	Fn	Pi	Pn	Pm	Cr	En	Ec	Sm	Smu	Ss	Lc	Sa	Total
Aa		0.96 *	−0.81	0.24	0.55		0.98 *	0.92 *	0.56			0.18					−0.34
Pg			−0.03	0.22	0.02	−0.04	0.12	0.22	0	0.57 *	−0.32	−0.3 *	0.14	0.71	−0.05		−0.07
Tf				0.11	−0.67 *	−0.13	−0.34	−0.12	−0.13	0.05	−0.49	−0.88 *	−0.29	0.11	−0.31		0.05
Td					−0.12	0.55	0.07	0.62 *	−0.09	0.78 *	−0.34	−0.33 *	−0.08	−0.17	0.4		−0.16
Fn						0.26	0.33 *	−0.1	0.24	−0.12	0.28	0.43 *	0.38 *	0.33	0.19		0.01
Pi							0.75 *	0.21	0.56 *	0.58		0.15	−0.16				0.36
Pn								0.23	0.4 *	−0.22	−0.14	0.15	−0.15	−0.26	−0.17		−0.23
Pm									0.22	−0.09	−0.32	−0.11	−0.08	−0.37	0.11		−0.12
Cr										−0.32	−0.22	0.12	−0.18	0.95 *	0.22		−0.14
En												−0.13	0.31		0.3		0.45
Ec												0.2	0.18		−0.23		−0.24
Sm													0.12	−0.2	0.24		0.04
Smu															0.01		−0.23
Ss																	−0.49
Lc																	−0.12
Sa																	
Total																	

Abbreviations: Aa, *Aggregatibacter actinomycetemcomitans*; Pg, *Porphyromonas gingivalis*; Tf, *Tannerella forsythia*; Td, *Treponema denticola*; Fn, *Fusobacterium nucleatum*; Pi, *Prevotella intermedia*; Pm, *Parvimonas micra*; Cr, C. *Campylobacter rectus*; En, *Eubacterium nodatum*; Ec, *Eikenella corrodens*; Sm, S. *Streptococcus mitis*; Smu, *Streptococcus mutans*; Lc, *Lactobacillus casei*; Sa, *Staphylococcus aureus*; Pn, *Prevotella nigrescens*; Ss, *Streptococcus sobrinus*; and * *p* < 0.05.

**Table 6:** Interspecies correlations in subjects with severe periodontitis.

	Aa	Pg	Tf	Td	Fn	Pi	Pn	Pm	Cr	En	Ec	Sm	Smu	Ss	Lc	Sa	Total
Aa		0.96 *	−0.81	0.24	0.55		0.98 *	0.92 *	0.56			0.18					−0.34
Pg			−0.03	0.22	0.02	−0.04	0.12	0.22	0	0.57 *	−0.32	−0.3 *	0.14	0.71	−0.05		−0.07
Tf				0.11	−0.67 *	−0.13	−0.34	−0.12	−0.13	0.05	−0.49	−0.88 *	−0.29	0.11	−0.31		0.05
Td					−0.12	0.55	0.07	0.62 *	−0.09	0.78 *	−0.34	−0.33 *	−0.08	−0.17	0.4		−0.16
Fn						0.26	0.33 *	−0.1	0.24	−0.12	0.28	0.43 *	0.38 *	0.33	0.19		0.01
Pi							0.75 *	0.21	0.56 *	0.58		0.15	−0.16				0.36
Pn								0.23	0.4 *	−0.22	−0.14	0.15	−0.15	−0.26	−0.17		−0.23
Pm									0.22	−0.09	−0.32	−0.11	−0.08	−0.37	0.11		−0.12
Cr										−0.32	−0.22	0.12	−0.18	0.95 *	0.22		−0.14
En												−0.13	0.31		0.3		0.45
Ec												0.2	0.18		−0.23		−0.24
Sm													0.12	−0.2	0.24		0.04
Smu															0.01		−0.23
Ss																	−0.49
Lc																	−0.12
Sa																	
Total																	

Abbreviations: Aa, *Aggregatibacter actinomycetemcomitans*; Pg, *Porphyromonas gingivalis*; Tf, *Tannerella forsythia*; Td, *Treponema denticola*; Fn, *Fusobacterium nucleatum*; Pi, *Prevotella intermedia*; Pm, *Parvimonas micra*; Cr, *Campylobacter rectus*; En, *Eubacterium nodatum*; Ec, *Eikenella corrodens*; Sm, S. *Streptococcus mitis*; Smu, *Streptococcus mutans*; Lc, *Lactobacillus casei*; Sa, *Staphylococcus aureus*; Pn, *Prevotella nigrescens*; Ss, *Streptococcus sobrinus*; and * *p* < 0.05.

**Table 7:** Association of severe periodontitis according to levels of target species.

	Levels	No. of Subjects	No. (%) with Severe Periodontitis	Crude OR (95% CI)	*p* Value	Adjusted OR (95% CI)	*p* Value
Aa							
	0	79	45 (57.0)	1		1	
	1	4	3 (75.0)	1.8 (0.3–18.9)	0.557	13.7 (0.9–389.2)	0.059
	2	4	4 (100.0)	6.8 (0.7–915.8)	0.111	1.6 (0.1–232.1)	0.750
Pg							
	0	24	5 (20.8)	1	-	1	-
	1	31	23 (74.2)	10.9 (3.1–39.0)	<0.001	3.3 (0.4–26.6)	0.271
	2	32	24 (75.0)	11.4 (3.2–40.6)	<0.001	1.1 (0.1–8.7)	0.942
Tf							
	0	23	14 (60.9)	1	-	1	-
	1	32	13 (40.6)	0.4 (0.1–1.3)	0.141	0.1 (0.0–1.1)	0.059
	2	32	25 (78.1)	2.3 (0.7–7.5)	0.169	3.7 (0.3–48.1)	0.319
Td							
	0	36	16 (44.4)	1	-	1	-
	1	25	14 (56.0)	1.6 (0.6–4.4)	0.375	5.3 (0.6–44.8)	0.129
	2	26	22 (84.6)	6.9 (2.0–24.0)	0.002	7.3 (1.1–47.4)	0.035
Fn							
	1	43	30 (69.8)	1	-	1	-
	2	44	22 (50.0)	0.4 (0.2–1.0)	0.062	1.0 (0.2–4.3)	0.969
Pi							
	0	64	37 (57.8)	1	-	1	-
	1	11	7 (63.6)	1.3 (0.3–4.8)	0.717	0.7 (0.1–7.6)	0.762
	2	12	8 (66.7)	1.5 (0.4–5.3)	0.568	0.5 (0.1–3.4)	0.504
Pn							
	0	16	9 (56.2)	1	-	1	-
	1	35	25 (71.4)	1.9 (0.6–6.7)	0.289	120.4 (5.3–2725.4)	0.002
	2	36	18 (50.0)	0.8 (0.2–2.5)	0.677	22.5 (2.0–260.6)	0.012
Pm							
	1	43	19 (44.2)	1	-	1	-
	2	44	33 (75.0)	3.8 (1.5–9.4)	0.004	4.4 (1.0–20.1)	0.057
Cr							
	0	14	7 (50.0)	1	-	1	-
	1	36	25 (69.4)	2.3 (0.6–8.1)	0.203	3.5 (0.5–27.3)	0.226
	2	37	20 (54.1)	1.2 (0.3–4.0)	0.795	1.6 (0.2–11.0)	0.628
En							
	0	70	38 (54.3)	1	-	1	-
	1	8	5 (62.5)	1.3 (0.3–6.1)	0.694	0.3 (0.1–2.0)	0.227
	2	9	9 (100.0)	16.0 (1.9–2097.0)	0.006	4.3 (0.4–609.5)	0.280
Ec							
	0	68	37 (54.4)	1	-	1	-
	1	9	6 (66.7)	1.7 (0.4–7.3)	0.490	2.6 (0.2–30.9)	0.441
	2	10	9 (90.0)	7.5 (0.9–62.8)	0.061	13.6 (0.5–380.9)	0.124
Sm							
	1	43	33 (76.7)	1	-	1	-
	2	44	19 (43.2)	0.2 (0.1–0.6)	0.001	0.1 (0.0–0.8)	0.024
Smu							
	0	29	17 (58.6)	1	-	1	-
	1	29	18 (62.1)	1.2 (0.4–3.3)	0.788	2.5 (0.4–18.3)	0.354
	2	29	17 (58.6)	1.0 (0.4–2.8)	1	0.4 (0.1–2.5)	0.316
Ss							
	0	81	47 (58.0)	1		1	
	1	3	3 (100.0)	5.1 (0.5–692.1)	0.205	1.6 (0.1–244.0)	0.755
	2	3	2 (66.7)	1.2 (0.2–13.7)	0.856	1.4 (0.1–193.5)	0.873
Lc							
	0	63	34 (54.0)	1	-	1	-
	1	12	11 (91.7)	9.4 (1.1–77.0)	0.037	1.4 (0.1–14.1)	0.795
	2	12	7 (58.3)	1.2 (0.3– 4.2)	0.780	0.3 (0.0–1.9)	0.192
Sa							
	0	68	48 (70.6)	1	-	1	-
	1	9	2 (22.2)	0.1 (0.0–0.6)	0.011	0.2 (0.0–2.0)	0.162
	2	10	2 (20.0)	0.1 (0.0–0.5)	0.006	0.4 (0.0–7.4)	0.525
Total							
	1	43	18 (41.9)	1	-	1	-
	2	44	34 (77.3)	4.7 (1.9–12.0)	0.001	1.4 (0.3–5.8)	0.673

Level 0 indicates polymerase chain reaction (PCR)-negative subjects. Level 1 indicates that the number of bacterial cells is less than the median number in PCR-positive subjects. Level 2 indicates that the number of bacterial cells is equal to or greater than the median number in PCR-positive subjects. Logistic regression analysis was performed after adjusting for known confounders: sex, age, and smoking history. Abbreviations: OR, odds ratio; CI, confidence interval; Aa, *Aggregatibacter actinomycetemcomitans*; Pg, *Porphyromonas gingivalis*; Tf*, Tannerella forsythia*; Td, *Treponema denticola*; Fn, *Fusobacterium nucleatum*; Pi, *Prevotella intermedia*; Pm, *Parvimonas micra*; Cr, *Campylobacter rectus*; En, *Eubacterium nodatum*; Ec, *Eikenella corrodens*; Sm, *Streptococcus mitis*; Smu, *Streptococcus mutans*; Lc, *Lactobacillus casei*; Sa, *Staphylococcus aureus*; Pn, *Prevotella nigrescens*; and Ss, *Streptococcus sobrinus.*

## Data Availability

The datasets generated during the current study are available from the corresponding author on reasonable request.
